# Transient Monoclonal Gammopathy Induced by Disseminated *Staphylococcus aureus* Infection

**DOI:** 10.1155/2012/607104

**Published:** 2012-11-29

**Authors:** Dimitrios Stoimenis, Christina Spyridonidou, Nikos Papaioannou

**Affiliations:** 1st Department of Internal Medicine, “Georgios Papanikolaou” General Hospital, Exochi, 570 10 Thessaloniki, Greece

## Abstract

Monoclonal gammopathy reflects a serological disorder suggesting a plasma cell dyscrasia or a B-cell abnormality. However, it may occasionally be encountered as a transient manifestation in the course of several diseases including infections. This is the first reported case of a transient monoclonal gammopathy IgG lambda light chain associated with a *Staphylococcus aureus* infection that was complicated with renal abscess and vertebral spondylodiscitis in a previously healthy 68-year-old male. We observed a complete resolution of the gammopathy within three months of medical treatment before the entire restoration of all clinical and laboratory findings. Many invasive and cost-intensive diagnostic procedures had preceded the exclusion of a malignancy. The clinical significance and the exact pathogenesis of transient monoclonality are poorly understood and remain a matter of speculation.

## 1. Introduction

Monoclonal proteins represent unimpaired or partial immunoglobulin molecules that are exuded by a single clone of plasma cells. Most of the times the presence of a paraprotein in the serum implies a plasma cell dyscrasia such as multiple myeloma, Waldenström's macroglobulinemia, cryoglobulinemia, and primary amyloidosis or it may derives from abnormal B cells that have not been differentiated in plasma cells just like in leukemias or lymphomas [1]. Finally, it may express a benign condition such as monoclonal gammopathy of undetermined significance (MGUS) that is regarded as an immunologic response without specific meaning. It is met in chronic inflammatory disorders including chronic liver, collagen, vascular, granulomatous, and infectious diseases [2]. In medical literature as far as infections are concerned the main pathogenic agents that have been associated with the occurrence of a transient monoclonal gammopathy are mainly viruses and various gram-negative bacteria. However, to the best of our knowledge, there are no reports correlating the presence of a transient monoclonal gammopathy with specific gram-positive bacteria. Here, we report a case of a transient monoclonal gammopathy type IgG lambda during a long-lasting infection by methicillin-susceptible *Staphylococcus aureus* that was complicated with renal abscess and vertebral spondylodiscitis in a previously healthy individual.

## 2. Case Presentation

A 68-year-old man was admitted to our department of internal medicine in June 2010 because of fever, gradually worsening low back pain and difficulty in rising. These symptoms started approximately two weeks before his admission. The patient had no significant medical record and he did not receive any chronic medication. From the recent medical history he mentioned a restorative dental procedure two months ago and a right shoulder tendonitis three weeks ago for which nonsteroidal anti-inflammatory agents per os were administered.

 On admission the patient was febrile up to 38.5°C and oligoanuric. He was subjected to laboratory tests (Table 1) that revealed acute renal failure, normocytic normochromic anemia, leukocytosis with neutrophilia, and highly elevated markers of inflammation. Urine analysis detected considerable hematuria and albuminuria.

**Figure 1:**
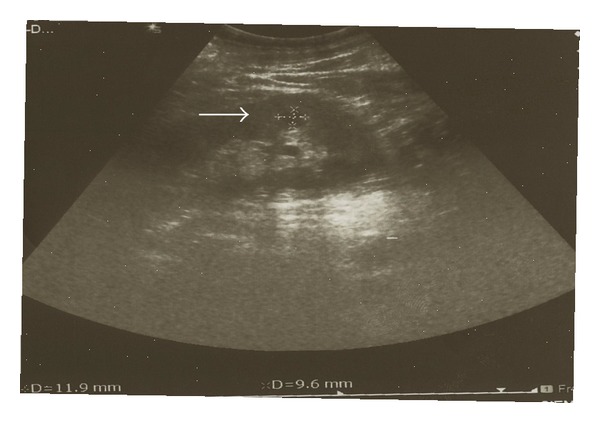
Ultrasound showing the renal abscess which appears as a hypoechoic area 1.19 × 0.96 cm within the cortex of the left kidney.

 The patient was started on a continuous intravenous infusion of furosemide and human albumin solution which resulted in a satisfactory diuresis. Besides, a renal ultrasound was performed, which disclosed a well defined hypoechoic area 1.19 × 0.96 cm within the cortex of the left kidney with low-amplitude internal echoes enhancement and lack of vascularity on doppler imaging (Figure 1). These findings were consistent with a renal abscess. Empiric therapy included meropenem 500 mg thrice daily iv (reduced dose on account of acute renal failure). Moreover, four blood and two urine cultures obtained after admission yielded methicillin-susceptible *Staphylococcus aureus* (*MSSA*). Endocarditis was excluded due to normal findings of transthoracic and transesophageal echocardiographs. One week later, de-escalation of antibiotic therapy was undertaken based on microbiological susceptibility data and teicoplanin 400 mg twice daily iv was initiated.

**Figure 2:**
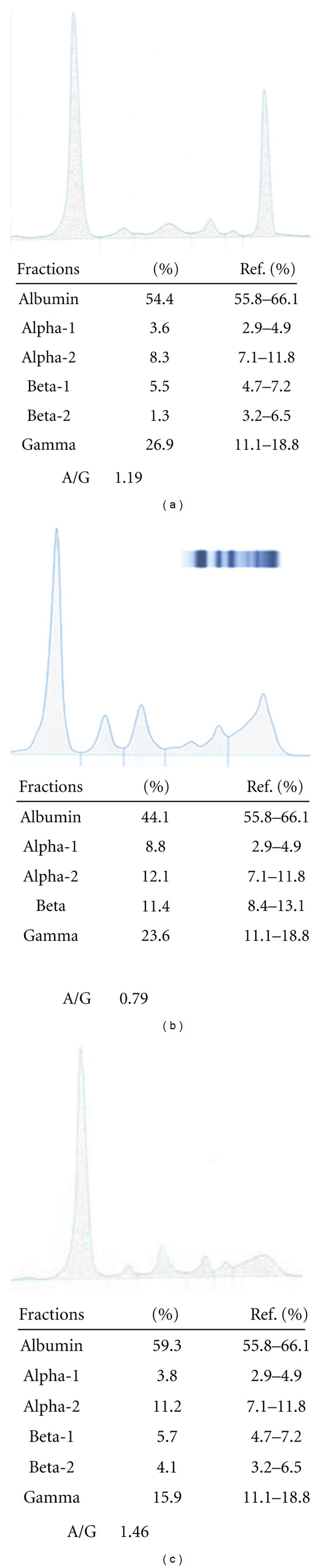
(a) Serum electrophoresis indicates a monoclonal component in the gamma region. (b) After two months a minor monoclonal component is detected. (c) Serum electrophoresis after three-month treatment shows normal albumin and globulin fractions.

The patient became afebrile on the third week and the renal function was progressively getting improved. Nevertheless, the deterioration of his anemia that imposed consecutive transfusions of RBC plus the persistence of the elevated markers of inflammation (ESR = 135 mm/h; CRP = 12 IU/L) raised the need for further examinations. A complete skeletal X-ray was initially performed but no lytic lesions were apparent. Serum calcium was normal, beta-2 microglobulin was slightly elevated (b2m = 3.50 *μ*g/mL) and urine albumin was 1.67 gr/d. Immunoglobulin levels were identified, showing an increase in IgG and normal IgA and IgM (Table 1). Serum electrophoresis showed a monoclonal component in the gamma region and the immunofixation electrophoresis (IFE) pattern demonstrated a typical paraprotein, IgG-lambda (Figure 2(a)). Bence Jones protein was not detected in urine analysis. The bone marrow aspiration revealed plasma cells to an extent of 6% and the bone marrow biopsy found only a few plasma cells (<10%). Lastly, flow cytometric immunophenotyping showed a normal population of T cells and a decrease of the B-cell population (2%, normal range 6–23%). Therefore, these results suggested most likely a MGUS rather than the likelihood of an underlying hematological malignancy.

**Figure 3:**
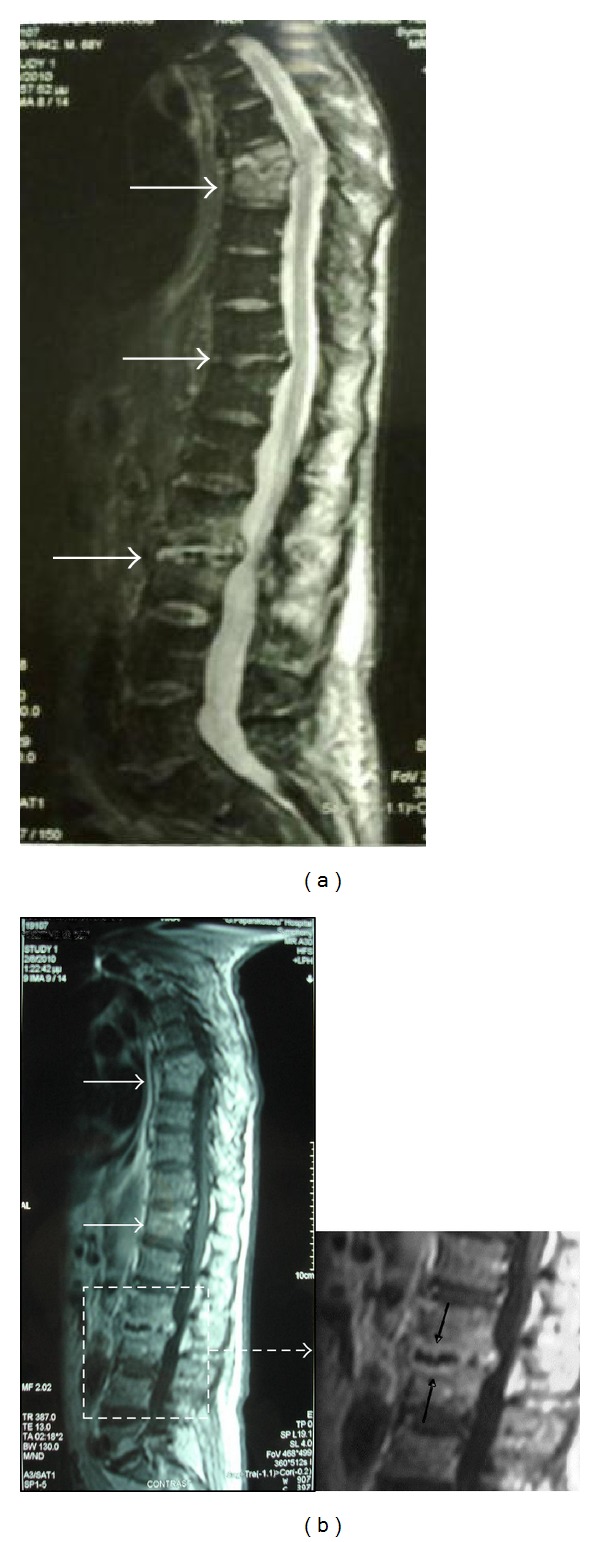
(a) Magnetic resonance imaging of thoracic and lumbar spine showing spondylodiscitis with a three-level involvement; T7-T8, T11-T12, and L2-L3 (white arrows). (b) A typical lesion is shown in the enlarged frame of the L2-L3 level; ellipsoidal contrast enhancement of the vertebral end-plates and the anterior aspect of the related disc space (black arrows).

Four weeks after admission the patient was afebrile and a renal ultrasound manifested a complete resolution of the abscess. Still, the patient complained of deterioration in back pain and at the same time the laboratory markers of inflammation were steadily elevated. Complete serological tests, neoplastic markers, Gen Probe and Ziehl-Neelsen stain for *mycobacteria*, Widal, Wright and Wright-Coombs as well as testing for HBV, HCV and HIV were all negative. Soon after the normalization of the renal function, a computed tomography scan with iv contrast of the abdomen, chest and spine was performed. The findings suggested a spondylodiscitis and diagnosis was confirmed by magnetic resonance imaging, which revealed a three-level involvement of the spine; T7-T8, T11-T12 and L2-L3 (Figure 3). 

The patient was treated for another two months with antistaphylococcal agents. Over this period the markers of inflammation gradually decreased while a second check of serum immunoelectrophoresis detected a minor monoclonal component (Figure 2(b)). After the three-month treatment no paraprotein was identified and the patient was discharged (Figure 2(c)). During the 2-year follow-up period the patient fully recovered to his previous ambulatory status and the laboratory tests returned to normal. 

## 3. Discussion

Monoclonal gammopathy of undetermined significance, albeit it is considered a benign condition, may as well signify a malignancy since it is known that 10% of those patients presenting MGUS will develop multiple myeloma within the next 20 years [3]. Its prevalence is assessed to vary from 0.05% to 6.10% according to Wadhera and Rajkumar [4]. In addition, the occurrence of MGUS increases with age. Ligthart et al. suggested that this correlation stands for immunodeficiency attributed to aging per se [5]. However the prevalence of MGUS in the general population is often underestimated as it is only investigated in patients with certain clinical symptoms. Accordingly, it is not clear if there is a true causation between certain diseases and MGUS or if it is all about a coincidental coexistence since the screening for monoclonality is biased [6].

As far as the transient MGUS is concerned it has been associated with various disorders. There have been reported mainly autoimmune diseases (i.e., SLE, autoimmune hepatitis, rheumatoid arthritis), drugs, and toxins [2]. Moreover, chronic infections like HBV and HCV, HIV, CMV, HSV, *Brucella*, *rickettsioses*, and *Bartonella* species have been illustrated as etiological agents [2, 7–11]. Although there is no established pathogenesis, it is assumed that patients suffered from diseases stimulating increased production of immunoglobulins over a long period may present a monoclonal gammopathy. 

In the aforementioned case the patient manifested a severe infection by *staphylococcus aureus*, normocytic anaemia, highly elevated ESR, acute renal failure, and a serum paraprotein in the intense course of the illness, which could have been explained by a single disease such as multiple myeloma. Besides, multiple myeloma is correlated with a vulnerability of the immune system to several bacterial infections [12]. However, the diagnostic criteria were not fulfilled and no haematological malignancy was documented. 

 After a three-month period of successful treatment with antistaphylococcal agents the analysis of serum immunoelectrophoresis revealed that the monoclonal protein had disappeared. All the manifestations were provoked by the severity and the duration of the staphylococcal infection posing thereafter a question whether this immunologic abnormality was triggered by specific staphylococcal antigens or by the chronic inflammation. Several works have demonstrated that *Staphylococcus aureus* infected osteoblasts in osteomyelitis induce IL-6 and IL-12 secretion [13]. IL-6 acts a growth factor for B-cell differentiation and terminal maturation into antibody producing plasma cells [14]. Thus, the inflammatory cytokines induced by the *MSSA* infection in our patient plausibly stimulated excess proliferation of the monoclonal immunoglobulin resulting in the development of MGUS.

In conclusion, this case illustrates that when a monoclonal component is detected in a patient suffering from a chronic bacterial infection, clinicians should consider the likelihood of a transient paraproteinemia and only if a malignancy is suspected or the monoclonality persists, should the paraproteinemia screening algorithms be followed. 

## Figures and Tables

**Table 1:** Laboratory findings of our patient at presentation and on discharge.

Laboratory value	Admission	Discharge	Normal range
Hematocrit	25.6	34.4	40.0–52.0%
Hemoglobin	8.7	11.3	13–17.8 g/dL
MCV	78.7	82.7	80–99 fL
Leukocytes	13.1	3.51	4.0–10 × 10^3^/*μ*L
Neutrophils	85.3	53.3	40–75%
Lymphocytes	7.3	29.3	20–45%
Platelets	326	221	150–450 × 10^3^/*μ*L
PT	17.7	12	10.5–12.5 sec
apTT	29.9	32.2	27–34 sec
Urea	232	32	14–50 mg/dL
Creatinine	11	1	0.7–1.4 mg/dL
Total proteins	6.1	6.1	5.5–8 g/dL
Albumin	2.3	3.5	3.5–5.5 g/dL
Globulins	3.8	2.6	1.5–3.5 g/dL
IgA	396	—	85–450 mg/dL
IgG	2600	—	800–1700 mg/dL
IgM	112	—	63–277 mg/dL
Procalcitonin	8.83	0	<0.30 ng/mL
ESR	130	22	1–10 mm/1st hour
C-reaction protein	13.2	0.99	<0.80 IU/L

Urinalysis on admission			

Erythrocytes >100 per HPF, Leukocytes = 3-4 per HPF			
Hemoglobin = 4+, protein = 3+, Nitrite = positive			
